# Barriers to and Facilitators of Technology in Cardiac Rehabilitation and Self-Management: Systematic Qualitative Grounded Theory Review

**DOI:** 10.2196/18025

**Published:** 2020-11-11

**Authors:** Shreya Tadas, David Coyle

**Affiliations:** 1 University College Dublin Dublin Ireland

**Keywords:** telemedicine, cardiovascular diseases, self-management, self-care, systematic review, grounded theory, mobile phone

## Abstract

**Background:**

Dealing with cardiovascular disease is challenging, and people often struggle to follow rehabilitation and self-management programs. Several systematic reviews have explored quantitative evidence on the potential of digital interventions to support cardiac rehabilitation (CR) and self-management. However, although promising, evidence regarding the effectiveness and uptake of existing interventions is mixed. This paper takes a different but complementary approach, focusing on qualitative data related to people’s experiences of technology in this space.

**Objective:**

Through a qualitative approach, this review aims to engage more directly with people’s experiences of technology that supports CR and self-management. The primary objective of this paper is to provide answers to the following research question: What are the primary barriers to and facilitators and trends of digital interventions to support CR and self-management? This question is addressed by synthesizing evidence from both medical and computer science literature. Given the strong evidence from the field of human-computer interaction that user-centered and iterative design methods increase the success of digital health interventions, we also assess the degree to which user-centered and iterative methods have been applied in previous work.

**Methods:**

A grounded theory literature review of articles from the following major electronic databases was conducted: ACM Digital Library, PsycINFO, Scopus, and PubMed. Papers published in the last 10 years, 2009 to 2019, were considered, and a systematic search with predefined keywords was conducted. Papers were screened against predefined inclusion and exclusion criteria. Comparative and in-depth analysis of the extracted qualitative data was carried out through 3 levels of iterative coding and concept development.

**Results:**

A total of 4282 articles were identified in the initial search. After screening, 61 articles remained, which were both qualitative and quantitative studies and met our inclusion criteria for technology use and health condition. Of the 61 articles, 16 qualitative articles were included in the final analysis. Key factors that acted as barriers and facilitators were background knowledge and in-the-moment understanding, personal responsibility and social connectedness, and the need to support engagement while avoiding overburdening people. Although some studies applied user-centered methods, only 6 involved users throughout the design process. There was limited evidence of studies applying iterative approaches.

**Conclusions:**

The use of technology is acceptable to many people undergoing CR and self-management. Although background knowledge is an important facilitator, technology should also support greater ongoing and in-the-moment understanding. Connectedness is valuable, but to avoid becoming a barrier, technology must also respect and enable individual responsibility. Personalization and gamification can also act as facilitators of engagement, but care must be taken to avoid overburdening people. Further application of user-centered and iterative methods represents a significant opportunity in this space.

## Introduction

### Background

Cardiovascular diseases (CVDs) are the leading cause of death worldwide. An estimated 17.9 million people died from CVD in 2016, representing 31% of all global deaths [1,2]. By 2035, more than 130 million adults in the US population (45.1%) are projected to have some form of CVD, with the total costs of CVD expected to reach US $1.1 trillion [3]. Improved cardiovascular outcomes depend largely on how well affected people manage their condition [4]. Physical rehabilitation and lifestyle management are critical components of programs aimed at primary and secondary prevention of CVD. A major challenge in implementing these strategies is ensuring good patient engagement and compliance with prescribed exercise programs and nutrition plans. Evidence from the literature suggests that tightly supervised intervention programs are most successful and that self-directed management is less successful because of problems with engagement and adherence. The problem lies in expecting patients with a wide variety of life patterns and personality types to conform to standardized programs that do not fit with their ever-changing context [5].

After a person is hospitalized and following a discharge and recuperation period, they are typically recommended to attend a cardiac rehabilitation (CR) program offered by hospitals. Following this, they need to continue to self-manage their cardiac health. CR is considered a vital part of long-term recovery by targeting risk factor modification, supervised exercise, psychological support, and medication review [6]. However, the uptake of CR programs remains poor because of factors such as age, gender, lack of knowledge, transportation, motivation, and social support [7,8]. This also has an impact on people’s subsequent ability to self-manage their condition. Barlow et al [9] state, “self-management refers to the individual’s ability to manage the symptoms, treatment, physical and psychological consequences and lifestyle changes.” Recent research suggests that digital health interventions can play an important role in supporting both rehabilitation and self-management. A systematic review of mobile phone apps to support self-care following heart failure by Athilingam and Jenkins [10] demonstrated positive trends and cost-effectiveness, enabling increased access to symptom monitoring and promoting patient engagement in their own homes. Similarly, a review by Piette et al [11] on mobile health (mHealth) technologies for CVD reduction and management found evidence that mHealth interventions can improve cardiovascular-related lifestyle behaviors and disease management. The authors emphasize the need for new interventions that build on evidence-based behavioral theories and are adaptive to a patient’s unique and changing needs. Jörntén-Karlsson et al [12] also suggested mHealth as an effective long-term alternative to face-to-face rehabilitation and consultation, with the potential to reach more patients at a relatively lower cost. They found evidence that digital interventions can have a positive impact on patients with CVD but again stressed the need for easy to use, personalized, and user-friendly apps that can cater to patients from all age groups, especially older age groups. This recognition of the specific needs of older adults is critical, given the significant impact of CVD among this age group. However, recognizing the potential of technology to support patients with CVD across diverse age groups is also important, given the evidence from Foster et al [13] and Andersson and Vasan [14] that CVD impacts adults in all age groups. In line with this, a survey conducted by Gallagher et al [15], to assess the use of mobile technology among different demographics, demonstrates that mobile technology, when modified to suit different subgroups, offers an important opportunity to improve access to secondary prevention for cardiac patients.

Although there is a significant literature and a growing number of reviews on digital interventions for CVD rehabilitation and management, most previous studies base their conclusions on quantitative data. To better understand what drives the effectiveness and usage of technologies, there is also a need to analyze the collective perspectives of patients, focusing on their experiences, needs, and the barriers they face in using digital interventions. The literature outlined earlier has provided evidence that personalization [16] and the application of appropriate theory will play an important role in improving digital health technologies that target CVD. For example, behavior change theories and models can help inform the design of technical systems, guide evaluation strategies, and define target users [17,18]. In addition, persuasive design patterns can be used in digital interventions to address the challenge of obtaining sustained user engagement and behavior change among patients with CVD [19]. Building on this evidence, a greater understanding of patients’ experiences will provide the insight needed to design future technology and increase the success of technologies when deployed in real-world contexts. By improving adherence to lifestyle changes, appropriately designed digital health technologies that apply this insight can ultimately help to prevent recurrence of cardiac conditions.

The analysis in this paper draws strongly on research in the field of human-computer interaction (HCI). Our findings are analyzed from an HCI perspective, which emphasizes the benefits of iterative development of technology and user involvement throughout the design and evaluation process [20-23]. HCI approaches have been successfully applied to rehabilitation and self-management in other health domains [24-27]. Our decision to focus on both rehabilitation and self-management followed multiple discussions among the authors and cardiologists, which reflected the degree to which these issues are interconnected. The papers selected in this review have dealt with some of the common issues and challenges. An overview of these interventions, along with the synthesis of patients’ experiences, can be beneficial to both medical and HCI researchers. To the best of our knowledge, no previous systematic review has combined qualitative review methods and an HCI perspective to identify challenges and opportunities in the design of technology to support CR and self-management.

### Objectives

The primary objective of this paper is to provide answers to the following research question: What are the primary barriers to and facilitators and trends of digital interventions to support CR and self-management? This question is answered by synthesizing evidence from both medical and computer science literature. Using a qualitative approach, we aim to engage more directly with people’s needs from and experiences of technology that supports CR and self-management. Given the strong evidence from the field of HCI that user-centered and iterative design methods increase the success of digital health interventions, we also assess the degree to which user-centered and iterative methods have been applied in the studies included in this review.

This review follows the grounded theory literature review (GTLR) method [28]. GTLR aims at producing new insights and enables researchers to develop concept-centric yet accurate reviews through a 5-stage iterative process. The GTLR method adopts a rigorous search and selection process, eventually invoking the grounded theory method for the analysis stage. GTLR recommends that initial research questions are identified at the outset of the review process and allows for a bottom-up iterative approach in which new concepts are identified via a thorough and progressive analysis. Initial questions help focus on the review during the selection and analysis stages, but based on concepts identified during the analysis stage, it is acceptable for the final concepts to differ somewhat in focus from the initial questions. Following multiple rounds of discussion and refinement among the authors and cardiologists involved in this project, the following initial research questions were identified:

1. What kind of technological support is provided for CR and self-management?
2. What design approaches were applied in designing the technologies identified?
3. What experiences and attitudes do patients have of technology?
4. What are the barriers to using technology for rehabilitation and self-management after a cardiac incident?
5. What are the facilitators for using technology for rehabilitation and self-management after a cardiac incident?

## Methods

### Overview

This review follows the 5 stages recommended in the GTLR method [28]: (1) identifying the key research questions, appropriate sources, and search terms; (2) search for potential papers; (3) defined filtering for selection of papers and refining the sample for review; (4) a comparative and in-depth analysis of the papers through 3 coding levels; and (5) representing the emerging categories and concepts. In addition, we used the Preferred Reporting Items for Systematic Reviews and Meta-Analysis (PRISMA) as guidance for conducting this review. The complete PRISMA checklist for this paper is included in Multimedia Appendix 1.

In this section, we describe the inclusion and exclusion criteria of our review, database sources and search keywords used, the screening and selection process, data extraction process, and, finally, the analysis process.

### Search Strategy

To include a wide range of perspectives on designing technologies for rehabilitation and self-management of cardiac conditions, we selected papers from PsycINFO, Scopus, PubMed, and ACM (Association for Computing Machinery) Digital Library. HCI literature about designing technology for cardiac conditions was gathered from the ACM Digital Library. Similarly, psychology and medical literature on these types of technologies were gathered from PsycINFO and PubMed. Other major journals and conferences, such as Biomed Central, IEEE (Institute of Electrical and Electronics Engineers), BMJ (British medical journal), International Journal of Telemedicine and Applications, SAGE (Scientific Advisory Group for Emergencies), and Global Telehealth, are included in Scopus.

Title, abstract, and keyword searches were carried out on the above mentioned databases to obtain the results for this review (Multimedia Appendix 2). On the basis of the studies we were familiar with and to follow a structured process to define the keywords, we selected keywords to address 3 areas: domain, technology, and intervention that we considered most relevant to identify papers of interest (Textbox 1). Domain keywords focused on CVD as the main field interest, together with related medical terms (eg, coronary artery). Technology keywords addressed diverse technologies used in inventions (eg, mobile phones, sensors, and telehealth). Intervention keywords reflect the different types of interventions addressing the field of CVD (eg, tracking and behavior change). It is important to note that our search strings include both Medical Subject Headings (MeSH) and non-MeSH terms. This decision was made because the study aimed at a broad exploration of research in both technology (eg, HCI and software engineering) and medical disciplines. The technology databases included in our study (eg, the ACM Digital Library) do not recognize MeSH terms. Including both MeSH and non-MeSH terms represented the most balanced approach and helped to ensure consistency of search terms across the different databases.

We limited our search to papers published in the last 10 years and focused on papers in the English language and including adult patients.

**Keywords used in the search terms.**

Domain

- Cardiovascular disease
- Cardiology
- Cardiac
- Heart disease
- Coronary heart disease
- Coronary artery disease
- Heart failure

Technology

- Mobile
- Wearable
- Wearable sensors
- mHealth interventions
- Smartphone
- Tele-monitoring
- Sensing system
- Telehealth
- Telemedicine

Intervention

- Persuasive or persuasion
- Quantified self
- Tracking
- Behavior change or behavior
- Personal informatics
- Habit
- Prevention
- Detection
- Rehabilitation
- Management

### Eligibility Criteria

The review was concerned with the use of technology for self-management and rehabilitation practices in the context of CVDs. This excluded several papers that would otherwise be featured in the review, such as those suggesting design concepts without evaluating them [29,30], those describing algorithms or software architectures to solve specific self-care problems [31,32], and those focusing on monitoring and detection techniques to support primary prevention of CVD [33,34]. These types of studies are very relevant to CVD in a broader sense, but as they do not provide evidence on the use of technology to support self-management or rehabilitation, they were excluded from the review. The papers included in this review involved an active role for patients living with cardiac conditions and technology that could be controlled by the patients rather than those in which patients have a more passive role. This meant excluding a number of technologies used only in clinical settings and technologies based on biomarkers, photoplethysmogram, implantable devices, and defibrillators. Excluding them enabled us to focus on the lived experience of people with CVD, rather than the clinical context of care.

Furthermore, this review focuses on studies of patients with cardiac conditions. This excluded self-management and rehabilitation technologies focusing on other chronic conditions [19], wellness and lifestyle [35,36], or quantifying habits for health [37,38]. By keeping the focus on cardiac conditions, the motivation for using the technology was to maintain cardiac health, not to pursue personal interest, leisure, or general well-being, which would likely bring different principles for design and use. To attain subjective perspectives of patients’ needs and seek answers to our research questions, we focused on qualitative study methods for this review. Therefore, to be eligible for inclusion in this review, papers needed to include a technology intervention for cardiac management or rehabilitation, use qualitative study methods, and describe the use and evaluation of technology with users. Papers that did not follow the criteria were rejected. The inclusion and exclusion criteria are listed in Textboxes 2 and 3, respectively.

**Inclusion criteria.**

Domain

- Cardiac condition

Technology

- Use of technology with evaluation
- Technologies having active patient role (eg, mobile, wearable, mobile health, and telemedicine)

Intervention

- Secondary prevention involving self-management and rehabilitation

**Exclusion criteria.**

Domain

- Other chronic conditions and general well-being and lifestyle

Technology

- Design concepts, technology description, algorithms, and software architecture without evaluation
- Technologies having passive patient role: biomarkers
- Technology used in clinical settings
- Photoplethysmogram
- Implantable devices
- Defibrillators

Intervention

- Detection and monitoring for primary prevention

### Screening and Data Extraction

The search keywords retrieved 4282 articles, of which 3973 remained after removing duplicates. We first performed a prescreening of these papers by reading the title and abstract and removed papers concerning research abstracts, systematic reviews, protocols, workshops, studies dealing with patients aged <18 years, studies involving chemical and biological sciences, and studies involving clinical procedures. At this stage, the first author (ST) reviewed all papers, and the second author was consulted in any situation where the first author was uncertain. Where any disagreement occurred, the paper was not excluded at this stage. In the second phase of screening, the first author reviewed the title and abstract of all remaining papers using the full eligibility checklist to decide if they should be included in preselection. This was done to exclude papers that involved studies inclined toward medical and clinical techniques, for example, studies related to biomarkers, photoplethysmogram, implantable devices, and defibrillators and studies related to algorithms, methods, and techniques. The second author reviewed 10% (170/1700) of the papers at this stage, and agreement was verified across both authors. Where any disagreement was found, the paper in question was reviewed again by both authors and discussed to reach an agreement. Both researchers then met and cross-checked 50% of the final preselection list, discussed inconsistencies, and agreed upon a final list that included 61 papers for potential inclusion.

**Figure 1:**
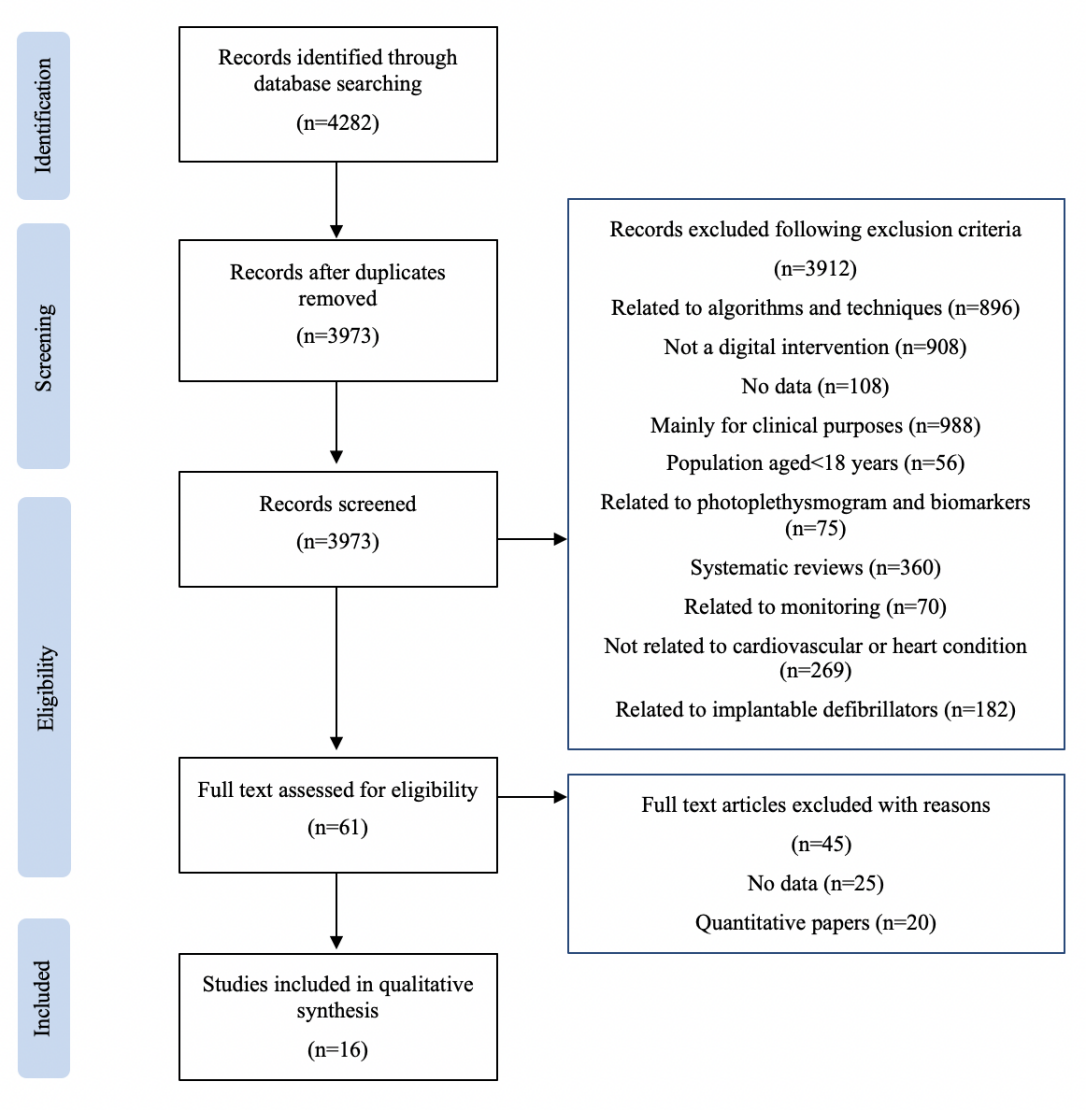
Flow diagram illustrating the screening and selection process of papers.

Each of these papers was further assessed in the final stage of the screening process to check if they applied qualitative methods and included qualitative data. Any paper that contained both quantitative and qualitative data was included in the final review, but only qualitative data in these papers were analyzed. A total of 25 papers were found to include no data, and 20 papers included only quantitative data. These papers were excluded. This left 16 papers that included qualitative data in our final analysis. Figure 1 provides an overview of the full screening process.

The critical appraisal skills program (CASP) checklist [39] was used to assess the quality of included studies and avoid the risk of bias. The CASP checklists are divided into 3 sections to assess internal validity, results, and relevance to the practice of published papers, and these sections are assessed by questions that can be answered with *yes*, *no*, or *can’t tell*. On the basis of the number of questions scored *yes*, an overall rating of *strong*, *moderate*, or *weak* was given to each study. The results of the assessment indicate that the majority of the papers included in the review are strong, whereas others are rated as moderate. Full details of the CASP assessment are included in Multimedia Appendix 3 [33,40-53].

Data from the included papers were initially extracted based on the keywords used in the search terms and eligibility criteria (Textboxes 1 and 2). This included data such as the number of participants, study methods, and settings for each study. In the final stage of data extraction, the full findings and discussion sections of each of the 16 papers were extracted. This provided data for our subsequent analysis.

### Analysis and Synthesis

The analysis step of the GTLR method involves a comparative analysis process with 3 levels of coding: open coding, axial coding, and selective coding. From the set of papers in the final review, ST selected a random paper and carefully read it again, highlighting principal findings, which the GTLR method calls excerpts. Similarly, excerpts from each paper were then listed. At the axial coding stage, these excerpts were articulated to form groups or insights. Both authors carried out an affinity mapping exercise on these excerpts. This led to the formation of groups and subgroups of the excerpts. At the selective coding stage, these groups were then compared and moved around, followed by discussions among the authors to form themes. This process involved iterative back and forth analysis between the excerpts and groups identified, in which stages were repeated and papers reread until a final consensus was reached. The coding process was supported by Boardthing [54], a web-based notice board software that allows individual and collaborative coding and analysis. The themes were repeatedly discussed and refined among the authors, and the analysis was only complete as the final version of the review documentation was ready.

## Results

### Study Characteristics

As noted earlier, the keyword search retrieved 4282 articles, of which 16 were included in the final analysis. An overview of the included studies is provided in Multimedia Appendix 4 [40-53,55,56].

#### Target Users

All studies in the final list focus on patients who had gone through or were going through a cardiac condition. Some of the studies specifically targeted patients diagnosed with heart failure, myocardial infarction, and coronary heart disease. Furthermore, some studies particularly involved participants’ postcardiac condition awareness and those who were in their CR phase. Some studies also involved physicians, informal caregivers, nurses, and cardiologists as participants. The papers included studies on both CR [45,46,50,51] and self-management [40-44,47-49,52,53,55,56].

#### Different Technology Support Provided

In general, the papers in this review investigated mobile or web apps, with some integrating sensors, to manage cardiac conditions. Papers featuring a web-based digital intervention were included [40,41,53]. Some studies used mobile [43,45-47], tablets [48], and a combination of web and mobile systems [42,44,49,50,55,56] as digital interventions. Overall, 2 studies did not involve any particular system. Instead, they focused on patients’ needs and perspectives of using an existing technology and the potential of digital interventions for cardiac management [51,52].

#### Motivation of the Studies

In general, support for self-management was provided through apps that aim to increase adherence, motivation, and engagement. These could be achieved through gamification [45], by providing guidance and education about the condition [40,43,47,52,55], through reminders and notifications, or by using patient data and sensor data to track and show their progress [46,49,52]. Many studies have involved interventions to increase physical activity and exercise for cardiac patients [41,44,46,48]. Studies also aimed to facilitate better connection between patients and care providers, nurses, or health professionals by providing a medium to communicate and share data [43,53,56]. Two papers were about virtual and remote CR to enable rehabilitation for patients in rural and distant locations [46,50]. One study focused on gathering the needs and interests of patients with CVD to effectively enable remote CR [51].

#### Design Approaches Used in the Studies

Table 1 provides an overview of the design methods and guiding theories used in the studies. Overall, as all the papers in the final list are qualitative studies, most of the papers used surveys, interviews, and usability tests and represented their evaluation and findings through themes (Table 1). Among these, some studies used theoretical frameworks of behavior change and user-centered design approaches and methodologies. Examples include scenario-based tests, card sorting, goal-directed design, and persuasive design [41-44,47,50,53,55,56]. One study used grounded theory to identify themes from participant responses [49]. Another study used gamification design principles to design the system with the aim of increasing motivation and adherence to lifestyle changes [45]. One study assessed the usability of technology using satisfaction surveys [48], another used a technology usage questionnaire to understand technology usage [51], and another used the system usability scale to assess the usefulness of a system [46].

**Table 1:** Overview of the theories and design approaches used in the final review.

Study	Design method or guiding theory	Users involvement
Dithmer et al [45]	Gamification and gameful design principles (PERMA^a^) are used to design the app. Gamification principles such as badges, levels, and leader boards were used to increase engagement and motivation.	Requirements gathering, design or prototyping, and evaluation or validation
Yehle et al [49]	No particular design principles or theory and design methodology mentioned.	Requirements gathering and evaluation or validation
Villalba et al [56]	Goal-directed design methodology is applied. A three-phase design process is used: conceptualization, implementation, and validation.	Requirements gathering and evaluation or validation
Jarvis-selinger et al [53]	Diffusion of innovation theory was used as the theoretical lens along with the current telehealth literature for sensitizing concepts. The study used a qualitative methodology, employing a constructivist approach.	Requirements gathering
Fischer et al [40]	Used common sense model of illness representation and showed visualization of body structure and behavior based on different symptoms through a web-based app.	Evaluation or validation
Pfaeffli et al [42]	A library of text and video messages were developed using self-efficacy theory framework and published exercise guidelines.	Requirements gathering, design or prototyping, and evaluation or validation
Katalinic et al [48]	No particular design principles or theory and design methodology mentioned.	Evaluation or validation
Antypas and Wangberg [41]	Different models of health behavior change are combined to form the tailoring algorithm. Tailoring is used as the theoretical framework. A methodological approach that is used to combine the user input and health behavior theory to develop a physical activity digital intervention for cardiac rehabilitation.	Requirements gathering and evaluation or validation
Geurts et al [44]	The prototype design was guided by 3 pillars: simplicity and ease of use, reduce fear and anxiety, and direct and indirect motivation. A human-computer interaction perspective is given by categorizing design decisions according to 3 pillars and show how these pillars resulted in concrete app features.	Requirements gathering, design or prototyping, and evaluation or validation
Buys et al [51]	No particular design principles or theory and design methodology mentioned.	Requirements gathering
Cornet et al [47]	Three frameworks guided the design process: Systems Engineering Initiative for Patient Safety (Version 2.0), Patient Work Framework, and user-centered design.	Requirements gathering, design or prototyping, and evaluation or validation
Banner et al [50]	No particular design principles or theory and design methodology mentioned.	Evaluation or validation
Baek et al [43]	No particular design principles or theory and design methodology mentioned.	Requirements gathering, design or prototyping, and evaluation or validation
Salvi et al [55]	Fogg’s Persuasive Systems Design principles were used when designing the GEx system, and health belief models were used to classify patients on the basis of the perceived benefits and barriers to self-efficacy in healthy behavior. The system design and development were guided by a combination of methodologies: Goal-Directed Design, Persuasive Systems Design, and agile software development. The desired behaviors were mapped into specific system’s specifications, borrowing concepts from Fogg’s Persuasive Systems Design principles.	Requirements gathering and evaluation or validation
Beatty et al [46]	No particular design principles or theory and design methodology mentioned.	Requirements gathering, design or prototyping, and evaluation or validation
Smith et al [52]	No particular design principles or theory and design methodology mentioned.	Requirements gathering

^a^PERMA: Positive emotion, Relationships, Meaning, and Accomplishment.

The 3 main stages of the HCI design process included in the ISO 9241 HCI development lifecycle are requirements gathering, producing design solutions, and evaluating the design against the requirements [57]. There is also a recommendation that this process is iterative, typically involving multiple cycles of design and evaluation. The design process, also known as the user-centered design, focuses on users and their needs in each stage of the process, and iteration continues until it is fit for implementation. We found limited evidence of studies applying a truly iterative approach and user-centered approach. A total of 9 of the 16 papers stated that a user-centered design approach was followed; however, it is not always clear that this involved multiple iterations of the design cycle [41-47,49,56]. Only 6 of the papers provided details of studies that involved users in each stage of the process [42-47]. Moreover, 3 of 16 studies involved users only in the final stage, that is, evaluation [40,48,50].

### Users’ Perspectives of Digital Interventions for Cardiac Self-Management and Rehabilitation

This section presents the final themes identified in our grounded theory analysis.

#### Knowledge

Evidence from the review suggests that knowledge plays an important role in rehabilitation and self-management. Education and knowledge influence self-management and increase confidence. To explain this further, we have categorized knowledge into 2 types: background knowledge and personal and in-the-moment understanding.

##### General Knowledge About CVD

General knowledge or background knowledge about CVD is the fundamental information or awareness that is required to be known by all patients with CVD. This can be information about one’s health condition, symptoms, body, medication, preventive measures, and advised lifestyle changes. Background knowledge also includes awareness about different support systems that help people to care for themselves, such as rehabilitation support and digital interventions.

There is a growing trend to use digital interventions to provide the required educational support. A study conducted to validate a self-care digital system to manage cardiovascular condition at home emphasized that education on symptoms and medication was highly valued by patients and health professionals; however, younger patients had reservations about lifestyle education, as they considered it to be intrusive and annoying. Similarly, patients who were initially scared of new technologies, later, after introductory explanations, found it easier to interact with the system [56]. Similarly, a study that evaluated the use of web-based visualizations of patient parameters to improve patients’ understanding of their disease and increase their level of control over the rehabilitation process shows that enhanced knowledge and understanding of the illness and its symptoms can motivate protective action, such as for individuals with heart failure to improve self-management of the illness and the symptoms [40]. For example:

Now I understand why my legs always swelled up.

40

We truly know how to, what is happening inside his heart, and why he’s getting all these symptoms. In the 2 years that we’ve been dealing with this illness, it’s so good to have it summarised up so that we know how to care for ourselves better.

40

Participants also repeatedly referred to the need to find the right answers either through an online forum or some kind of knowledge bank [41]:

It should be a forum where you have the opportunity to get the right answers, access to a resource, this is what I believe it becomes. It has an effect.

41

CR classes are also popularly known to provide essential knowledge, guidance, and support for patients:

...Your class (cardiac rehabilitation) because they stressed what is really bad for you and what is good for you so that makes you stop and think when you are even buying your groceries to make sure you are getting the right stuff.

49

##### Personal and In-the-Moment Understanding

Personal and in-the-moment understanding is the supplementary information that patients seek to enhance their self-care process. This type of information is acquired through personal tracking and monitoring and refers to the ongoing knowledge people develop about their individual condition. Knowing one’s body plays a key role is achieving control of the cardiac condition; however, it may be difficult to notice some changes and trends in everyday life. Technology has been used to make health and contextual information more easily available to patients and caregivers on an ongoing basis [23]. Patients state that being monitored by technology increases their feeling of security and comfort by enabling a better ongoing understanding of their health [56]. Self-care technologies that use monitored data to guide people to exercise or train within recommended or safe zones boosted confidence and increased motivation:

The application is not only beneficial for people who are afraid to exercise, but also supports people that have a higher risk to train too much.

44

A study conducted to understand the current technology usage of patients with CVD and to understand their needs and interests found that ongoing advice on exercise ideas, exercise prompts, information on local exercise opportunities, healthy meal ideas and recipes, and practical ideas to manage stress received the highest ratings for inclusion in a technology-based CR platform [51]:

I am unsure if I am doing the right thing, like food, so I like advice on that.

52

#### Social Versus Individual

Although most patients often manage their care autonomously, clinicians, other people living with the same condition, and caregivers play an equally important role.

##### Individual Responsibility

Responsibility for change in behavior is personal [41]. Changing behavior is easier if new habits are created by replacing old bad ones. To retain changes, it is important to make it part of the daily routine. Ubiquitous technology can support behavior change in the challenging situations of everyday life and remind users of their own commitments:

If you could get a message every day, there and then?

41

I believe that someone gets used to it, if we make a system, habits. That it doesn’t get too much, that we know that...we go online...and we get our own responsibility of our own training.

41

Technology can support small personal achievements such as getting out of the house to get fit. The use of digital systems as a tool for self-management is valued, especially among the younger ones:

It gave me the opportunity to get out of the home and try and get myself fit after the operation. I believe it has achieved that and more. I feel better in myself and I can achieve most jobs without taking about it.

55

##### Connecting With Others

Patients often seek to connect with others living with the same condition, and they use these interactions to understand how to live with their condition, validate their assumptions about their body and self-care, and obtain emotional support [58]. A CR session is an excellent example of this type of environment. A theme repeatedly expressed by the patients of the CR program was the importance of not being alone in the rehabilitation and self-management process. This was an important factor that helped them during their visits to the rehabilitation center, and it was something they wished to maintain after their discharge [41]. In addition, CR attenders found great value in being able to ask nurses, cardiologists, and dietitians questions according to their specific needs [42]. Digital interventions also provide easy access to others with the same condition, health professionals, and experts. A study on the experiences of patients undergoing virtual cardiac rehabilitation program (vCRP) demonstrated the potential of vCRP as a medium to provide easy access to health care professionals, nurses, exercise specialists, and dieticians. Although there were some concerns about trust and privacy [41], many of the participants explained that having ongoing monitoring from health care providers as well as support for self-management activities helped them adhere to their recommended program:

You know I had stents four years ago, and you start off with the best of intentions, but nobody looks over your shoulder and you peter out. At this time, I felt this is a nifty program...somebody’s watching it and I better do it. Keeps you honest, keeps you focused.

50

Keeping in touch with the group helps to lift people’s mood, is comforting, and provides support; therefore, many patients liked to use forums and web-based groups. Groups and forums on the internet are seen to help individuals be more committed to fitness by sharing goal completions and bragging about it for healthy competition. Forums brought more focus and motivation, as it makes individuals feel obliged to do activities. A study that used gamification for telerehabilitation program of patients with CVD also demonstrates the importance of social and family support, with patients stating that the most important aspect of the game was being able to play with a partner, thus enabling them to deal with rehabilitation as a team:

Training diary on the Internet...And also have a group where someone can subscribe to a forum, or have a...to brag...yesterday I walked for an hour and today I have been to the training...and tomorrow I have thought, yes...So, it is like this that someone gets to, a bit, a bit like a competition, internally between each of us. We will train, as much as possible we will commit to ourselves a bit more also.

41

I am saying that if we have it fixed, one time per week, that we send a message to each other and then, then you feel committed to say yes, for as long as you like...Yes, then you must have something else that really, you have something else that you have to do, or else...you just do it.

41

#### Motivation and Demotivation

The systems in the listed papers took a number of approaches to provide engagement and motivation toward self-management. Some of the key features of technology and patients’ attitudes toward them are described below.

##### Feedbacks and Reminders

Digital health interventions such as text messages and mobile- and web-based app reminders push patients to maintain the desired changes [42]. Apps using gamification principles are considered motivating, as they allow score, activity and goal comparison, healthy challenges, and competitions. Creating small manageable tasks was positively received by heart patients. Apps use data visualizations to show meaningful comparisons and to see how well they progressed [45]:

I went cycling without the application today, but it was less fun!

44

Two teams explicitly stated that on a day with bad weather, they would not have gone for a walk had they not been motivated by the application.

45

Reminders in any form were positively accepted by the patients. Text messages, although intrusive, pushed them to perform exercises, and many stated that reminders such as an alarm are needed for medication management [56]. On the other hand, some patients did not like reminders, as they constantly reminded them of their sickness.

##### Tracking and Monitoring

Digital health interventions that had the ability to track patients’ activities, heart rate, and current health status and showed their progress over time were considered valuable and engaging [44]. In a study to understand the current technology usage of patients with CVD, 68% of patients reported that heart rate monitoring was important when exercising at home [51]. In addition, patients also anticipated that they would be able to manage their disease more efficiently if their daily data could be easily entered in an app and shared with their doctors [43]:

I like the fact that I can put all of that and track it, and that my doctors can as well. I can show my doctor what I’ve been working on.

46

I think that the idea of an app that records all of the information that this app is doing will be very valuable. Actually somewhat of a motivation for me to do this thing.

46

##### Personalization

Some studies in this review suggested that digital interventions that gave the user the ability to personalize the app based on personal interests contributed toward motivation [41,44]. For example, one of the patients in a study that evaluated patients’ motivation when using a mobile app that guided them while cycling suggested that the app would be more engaging and fun if it had the flexibility to insert his preferred routes along with the preloaded ones. However, another patient in the same study preferred predefined routes [44]. Another study showed that although patients preferred simple interaction methods, they also asked for the possibility of applying advanced settings [51]. The findings of the same study also suggested that the future of technology-enabled CR might include different solutions to reach both men and women to better engage a broader target population of patients with CVD [51].

##### Increased Burden

Some studies in this review demonstrated patients’ concerns regarding using technology. For instance, some patients suggested that adding a device on top of what they already have led to them getting side tracked and thus not using it every day [47]. Patients in the older age group were especially resistant to use technology; some of them lacked interest and found it burdensome:

I’m retired and I gave all the computerization that I wanted up, that is it I do not even look at it and I will not even turn it on.

53

Furthermore, lack of time and other priorities is a barrier to self-management and use of technology. Most patients already have measuring devices at home, such as weight scales and blood pressure cuffs, and preferred to continue using devices they already know [56]:

There are people who like this (application) kind of stuff...and got the time. So for these people it might be great.

47

##### Acceptability of Technology

In contrast, studies in this review also demonstrated patients’ willingness to use technology. For example, one study reported that patients’ interest or intent to use an app for CVD management was high, despite the fact that most were older people who were unfamiliar with the information technology environment [43]. Overall, in most studies, patients as well as clinicians readily accepted and showed interest in learning about new technology [43,48].

Nevertheless, to reach the entire target population of patients with CVD, a variety of technology solutions should be designed to reach both men and women [51].

##### Usability

Finally, usability and ease of use are crucial for the acceptance of any type of digital intervention and thereby influence engagement. Many studies in this review emphasize that simple interaction methods are preferable. For example, one study stated that 38% of the patients preferred an interaction of no more than a few mouse clicks [51]. Patients unfamiliar with technology positively stated that it was just a matter of getting accustomed, and if they learned and used the app regularly, they would find it simple. Some patients also suggested considering e-literacy issues and initial training [41]:

It was pretty easy...I like that it’s simple.

46

I’m not used to this. Once I get used to it, I’ll know where everything is.

46

## Discussion

### Principal Findings

This review aims to understand users’ perspectives of technology in CR and self-management and identify barriers and facilitators of the use of technology. The results suggest that many patients have a positive attitude toward the use of technology. The grounded theory approach enabled us to identify common themes across the included papers, resulting in 3 principal findings:

1. Designers of new technologies and clinicians recommending existing systems to patients should consider seeking the support of both background knowledge and greater in-the-moment understanding. Background knowledge and awareness about the condition and its symptoms, medication, and posthospital care measures are important factors for effective self-management. However, effective self-management also requires patients to be aware of their current body condition and changes in their body, providing reassurance and enabling them to take appropriate measures in self-management.
2. Self-care is a personal responsibility, and people like to try different ways to keep themselves motivated to continue performing self-management activities. For some, but not all, opportunities to stay connected with family, caregivers, and others with a similar health condition are considered as one of the most effective ways to stay motivated and driven toward rehabilitation activities. Again, technology that supports both approaches is likely to be most beneficial.
3. Technologies can use different approaches to support engagement and motivation toward rehabilitation and self-management, including personalization, tracking and monitoring, reminders, and feedback. However, they should take into account the potential to demotivate because of issues including overburdening caused by different devices and apps, privacy concerns, lack of trust, lack of interest, and system usability. If not properly accounted for, these issues can impact the acceptability of systems and become major hindrances to effective rehabilitation and self-management.

These principal findings are discussed in greater detail below and also considered via the lens of relevant HCI literature.

Our first principal finding emphasizes the importance of different types of knowledge. Awareness of available resources, such as awareness of rehabilitation classes, existing online support groups, existing self-care digital apps, and remote rehabilitation videos and programs, is important so that patients can leverage these resources for better and sustained recovery and smoother transition to long-term self-management. In addition, ensuring that patients have knowledge of available emotional and physical support helps to foster self-efficacy if they feel overwhelmed by their CVD condition, leading to the inability to effectivity self-manage [59]. Prior work in HCI has also identified knowledge as an important factor influencing self-care. For example, a study exploring patients’ transition from hospitalization to self-management emphasizes gaps in knowledge, resources, and self-efficacy after discharge and demonstrates an interconnection between them [59]. The study describes knowledge as information provided to patients about their condition, medication, and management and resources as social and physical resources, for example, caregivers and access to health services. Self-efficacy is described as the patient’s confidence in their ability to self-manage their condition. The gaps highlighted in that study are consistent with the principal findings of this review. The authors recommend that at a system or hospital level, emphasis on verbal communication of information should be avoided. Ubiquitous computing and embedded technologies could be used to capture and retain verbal information received during hospitalization. In addition, hospitals should provide support and trusted sources of information for patients’ access to expertise. On the basis of our findings, these recommendations are also clearly applicable to CR. Similarly, work in HCI describes how patients’ understanding of their illness and availability of social and physical resources mediate their self-efficacy [60]. In contrast with prior work, our study has also highlighted the importance of supporting in-the-moment knowledge, which can be acquired through tracking and monitoring. It appears that both types of knowledge can be an integral part of effective CR and self-management.

Effective self-management requires patients to change certain behaviors. An individual’s inclination to change behavior depends on the extent to which they are motivated to change [61,62]. Our findings highlight that motivation for action is driven by both individual factors, such as personal responsibility, emotions, and goals, and external influences, such as friends, family, caregivers, health professionals, and personalized and persuasive features of technology. These findings reflect on Deci and Ryan’s [61] self-determination theory of motivation, which states that a human’s optimal move toward growth is driven by 3 needs: autonomy, the need to have control over one’s behavior; relatedness, the need to interact or be connected to others; and competence, the need to experience positive effects of one’s activity. Previous HCI research [24,63] provides helpful guidance on how technologies can support these basic needs and also highlights design-related tensions that can arise in balancing different needs. For example, Nunes et al [24] highlighted tensions in the degree of autonomy to be provided to patients, noting that technologies should take into consideration the different levels of autonomy given to the patients for self-care, as it is highly dependent on the disease and the patient’s current condition. Although patients are in charge of their health condition, it is important to reflect on the stages or decisions where a clinician’s support is needed. Treatment of CVD relies on a combination of medication and lifestyle changes, and there exists an individual difference in the disease management process. Individual differences refer to how people are similar or different in their ways of thinking, feeling, and behaving [64]. This would include patient demographics, situational or contextual changes, and environment. The transtheoretical model of behavior change [65] suggests that effective behavior change could be obtained if personalized feedback with different motivational levels or at different stages of the behavior change process is provided to people. Therefore, it is important to take these differences into account and leverage technology to provide tailored care. In the case of health care technologies, the one-size-fits-all approach could hamper effective self-care practices [66,67]. Nunes et al [24] also stressed on integrating self-care technologies in everyday lives by prioritizing the lived experiences of patients. This is also emphasized in discussion of *lived informatics* and *design for interweaving* by Rooksby et al [68]. In other words, for health care technologies to be successfully integrated into an individual’s life, it is necessary to acknowledge the everyday life of the individual [5]. Moreover, the results of this review demonstrate that patients’ adherence to self-management through health care technologies can be improved if technology does not act as a burden in their daily life and is easy to use.

Digital health interventions draw on 2 central domains of study, those originating in health (eg, medicine, biomedical sciences, and psychology) and in technology disciplines (eg, computer science, HCI, and software engineering). This trend is seen in the papers listed in this review. Blandford et al [69] highlighted 7 areas of contrast in practice between technical and health research. They emphasize that skipping over stages of iterative design before investing in large-scale evaluation of digital health technology leads to suboptimally designed solutions. In the HCI community, there is a growing practice of involving end users early on in the design stage and then throughout the full design and evaluation process. In contrast, the studies listed in this review show limited evidence of applying user-centered and iterative design processes. Blandford et al [69] also suggested that failing to learn how the nuances of design affect user interaction and engagement leads to failure in replicating it in different contexts and propagates risk from one design to another. Future research on technology to support CVD should address these limitations. Involving relevant users, in this case, patients, caregivers, and health professionals, in each stage of the design process will help reduce user experience challenges and increase acceptance, leading to more effective digital health interventions. Core to addressing this limitation is appropriate and focused engagement with key patient groups. In this context, although CVD impacts adults across all age groups, it is important to also recognize that CVD and other chronic illnesses are particularly prominent among older populations, and their distinct challenges and complex needs have important implications for the design of such systems [70]. The effectiveness of user-centered design with older adults can be seen in the increasing number of studies involving older populations in the early design stages [71,72].

### Limitations

As the aim of this review is to investigate and obtain subjective evidence of the barriers and facilitators of using technology for CR and self-management, only qualitative papers were considered, and review was limited by the analysis of the included studies. The possibility of subjectivity in analyzing the findings is acknowledged, although strategies to limit bias were undertaken through the process of grounded theory analysis and consultation with a second reviewer. In addition, the included studies had varied sample sizes, and the technology was used for different amounts of time in different studies. We acknowledge that this variation could have had an impact on the themes emerging in this review.

### Reflective Statement by Authors

This research was conducted in the Republic of Ireland. It is part of the Eastern Corridor Medical Engineering (ECME) collaborative research project, which seeks to improve cardiovascular health with a broad focus on enhancing user-ready sensor technology; improving smart wearables; reducing the complexity of point-of-care diagnostics; and improving smart, clinically relevant monitoring in the assisted living and rehabilitation environments. ECME is a partnership between 5 academic research centers in Northern Ireland, the Republic of Ireland and Scotland, and the Southern Health & Social Care Trust. It involves collaboration between researchers in the medical and technology fields. Both the authors of this paper are based at the Insight Centre for Data Analytics at Dublin when this study was conducted. ST was raised in India and had lived in Dublin for 2 years at the time of the study. She has experience in User Experience design in mobile and assistive technologies. DC has multigenerational roots in Ireland and is an expert in the field of HCI with a focus on the design of digital health technologies. None of the authors have direct lived experience of CVD. This study did not seek to directly address issues such as ethnicity, social and cultural background, and gender, and standard checklists, including the CASP tool, were used to assess the quality of included studies. However, we recognize the potential for bias, both in its own analysis and in the original research papers.

### Conclusions

The primary objective of this review was to apply qualitative methods to answer the following research question: What are the primary barriers to and facilitators and trends of digital interventions to support CR and self-management? Our findings show that the use of technology is acceptable to many people undergoing CR and self-management. Although background knowledge is an important facilitator, technology should also support greater ongoing and in-the-moment understanding. Connectedness is valuable, but to avoid becoming a barrier, technology must also respect and enable individual responsibility. Personalization and gamification can also act as facilitators of engagement, but care must be taken to avoid overburdening people. The findings also highlighted the limited use of iterative, user-centered approaches to guide design in this space. Going forward, further application of user-centered and iterative methods represents a significant opportunity.

## Appendix

Multimedia Appendix 1 Preferred Reporting Items for Systematic Reviews and Meta-Analysis (PRISMA) checklist.

Multimedia Appendix 2 Search strategy.

Multimedia Appendix 3 Qualitative assessment table.

Multimedia Appendix 4 Table with overview of included studies.

## Abbreviations

CASP: critical appraisal skills program
CR: cardiac rehabilitation
CVDs: cardiovascular diseases
ECME: Eastern Corridor Medical Engineering
EU: European Union
GTLR: grounded theory literature review
HCI: human-computer interaction
MeSH: Medial Subject Headings
mHealth: mobile health
PRISMA: Preferred Reporting Items for Systematic Reviews and Meta-Analysis
vCRP: virtual cardiac rehabilitation program
