# Supplementary material for: Barriers to and Facilitators of Technology in Cardiac Rehabilitation and Self-Management: Systematic Qualitative Grounded Theory Review
Source: J Med Internet Res. 2020 Nov 11;22(11):e18025. doi: 10.2196/18025 (PMC7688378; doi:10.2196/18025)
Supplement: Multimedia Appendix 2 [file jmir_v22i11e18025_app2.pdf]

Multimedia Appendix 1: Search strategy

ACM	recordAbstract:(mobile OR wearable OR mhealth OR sensing OR tele* OR smartphone) AND recordAbstract:(cardiac OR coronary OR cardiovascular OR "heart failure" OR "heart disease") AND recordAbstract:(behavio*r OR "health*care" OR prevention OR detection OR rehabilitation OR persuasion OR persuasive OR informatics OR tracking OR monitoring OR quantified OR habit) OR acmdlTitle:(mobile OR wearable OR mhealth OR sensors OR "tele-monitoring" OR telehealth OR smartphone OR coronary OR "heart failure" OR "heart disease" OR cardiovascular OR behavio*r OR persuasive OR persuasion OR informatics OR tracking OR quantified OR prevention OR rehabilitation OR monitoring OR habit OR detection) OR keywords.author.keyword:(mobile OR wearable OR mhealth OR sensors OR "tele-monitoring" OR telehealth OR smartphone OR coronary OR "heart failure" OR "heart disease" OR cardiovascular OR behavio*r OR persuasive OR persuasion OR informatics OR tracking OR quantified OR prevention OR rehabilitation OR monitoring OR habit OR detection)	88
Scopus	ABS (mobile OR wearable OR mhealth OR sensors OR "tele-monitoring" OR telehealth OR smartphone) AND ABS (coronary OR "heart failure" OR "heart disease" OR cardiovascular) AND ABS (behavio*r OR persuasive OR persuasion OR informatics OR tracking OR quantified OR prevention OR rehabilitation OR monitoring OR habit OR detection) OR AUTHKEY((mobile OR wearable OR mhealth OR sensors OR "tele-monitoring" OR telehealth OR smartphone) AND (coronary OR "heart failure" OR "heart disease" OR cardiovascular) AND (behavio*r OR persuasive OR persuasion OR informatics OR tracking OR quantified OR prevention OR rehabilitation OR monitoring OR habit OR detection)) OR TITLE(mobile OR wearable OR mhealth OR sensors OR "tele-monitoring" OR telehealth OR smartphone OR coronary OR "heart failure" OR "heart disease" OR cardiovascular OR behavio*r OR persuasive OR persuasion OR informatics OR tracking OR quantified OR prevention OR rehabilitation OR monitoring OR habit OR detection) AND (PUBYEAR > 2007) AND (LIMIT-TO (LANGUAGE,"English "))	2862
PsycINFO	(AB(mobile OR wearable OR mhealth OR sensing OR "tele-monitoring" OR telehealth OR smartphone) AND AB(cardiac OR coronary OR cardiovascular OR "heart failure" OR "heart disease") AND AB(behavio*r OR "health*care" OR prevention OR detection OR rehabilitation OR persuasion OR persuasive OR informatics OR tracking OR monitoring OR quantified OR habit)) OR (TI(mobile OR wearable OR mhealth OR sensors OR "tele-monitoring" OR telehealth OR smartphone) AND TI(coronary OR "heart failure" OR "heart disease" OR cardiovascular) AND	102

	TI(behavio*r OR persuasive OR persuasion OR informatics OR tracking OR quantified OR prevention OR rehabilitation OR monitoring OR habit OR detection)) OR (IF(mobile OR wearable OR mhealth OR sensors OR "tele-monitoring" OR telehealth OR smartphone) AND IF(coronary OR "heart failure" OR "heart disease" OR cardiovascular) AND IF(behavio*r OR persuasive OR persuasion OR informatics OR tracking OR quantified OR prevention OR rehabilitation OR monitoring OR habit OR detection))	
PubMed	((mobile[Title/Abstract] OR wearable[Title/Abstract] OR mhealth[Title/Abstract] OR sensing[Title/Abstract] OR "tele-monitoring"[Title/Abstract] OR telehealth[Title/Abstract] OR smartphone[Title/Abstract]) AND (cardiac[Title/Abstract] OR coronary[Title/Abstract] OR cardiovascular[Title/Abstract] OR "heart failure"[Title/Abstract] OR "heart disease"[Title/Abstract])) AND (behavio*r[Title/Abstract] OR "health*care"[Title/Abstract] OR prevention[Title/Abstract] OR detection[Title/Abstract] OR rehabilitation[Title/Abstract] OR persuasion[Title/Abstract] OR persuasive[Title/Abstract] OR informatics[Title/Abstract] OR tracking[Title/Abstract] OR monitoring[Title/Abstract] OR quantified[Title/Abstract] OR habit[Title/Abstract]) Filters: in the last 10 years, Humans	1230
