# Supplementary material for: Barriers to and Facilitators of Technology in Cardiac Rehabilitation and Self-Management: Systematic Qualitative Grounded Theory Review
Source: J Med Internet Res. 2020 Nov 11;22(11):e18025. doi: 10.2196/18025 (PMC7688378; doi:10.2196/18025)
Supplement: Multimedia Appendix 3 [file jmir_v22i11e18025_app3.pdf]

Multimedia Appendix 2: Critical Appraisal Skills Programme Qualitative Checklist[1]

Quality Assessment for Qualitative Studies Table

1. Was there a clear statement of the aims of the research?
2. Is a qualitative methodology appropriate?
3. Was the research design appropriate to address the aims of the research?
4. Was the recruitment strategy appropriate to the aims of the research?
5. Was the data collected in a way that addressed the research issue?
6. Has the relationship between researcher and participants been adequately considered?
7. Have ethical issues been taken into consideration?
8. Was the data analysis sufficiently rigorous?
9. Is there a clear statement of findings?

Name (year)	1	2	3	4	5	6	7	8	9	Rating
Dithmer M. et al. (2016)	Yes	Yes	Can't tell	Yes	Yes	Can't tell	Yes	Can't tell	Yes	Moderate
Yehle KS. et al. (2012)	Yes	Yes	Yes	Yes	Yes	Can't tell	Yes	Yes	Yes	Strong
Villalba E, et al. (2009)	Yes	Yes	Yes	Yes	Yes	Yes	Can't tell	Can't tell	Yes	Moderate
Jarvis-selinger S. et al. (2011)	Yes	Yes	Yes	Yes	Yes	No	Yes	Yes	Yes	Strong
Fischer S. et al. (2011)	Yes	Yes	Yes	Yes	Yes	Can't tell	Yes	Can't tell	Yes	Moderate
Pfaeffli L. et al. (2012)	Yes	Yes	Yes	Yes	Yes	Can't tell	Yes	Yes	Yes	Strong
Katalinic O, et al. (2013)	Yes	Yes	Can't tell	Yes	Yes	Yes	Can't tell	Yes	Yes	Moderate
Antypas K. and Wangberg SC. (2014)	Yes	Yes	Yes	Yes	Yes	Yes	Can't tell	Yes	Yes	Strong
Geurts E. et al. (2016)	Yes	Yes	Yes	Yes	Yes	Can't tell	Yes	Yes	Yes	Strong
Buys R. et al. (2016)	Yes	Yes	Yes	Yes	Yes	Can't tell	Yes	Yes	Yes	Strong
Cornet VP. et al. (2017)	Yes	Yes	Yes	Yes	Yes	Can't tell	Yes	Yes	Yes	Strong
Banner D. et al. (2015)	Yes	Yes	Yes	Yes	Yes	No	Yes	Can't tell	Yes	Moderate
Baek H. et al. (2018)	Yes	Yes	Yes	Yes	Yes	Yes	Yes	Yes	Yes	Strong
Salvi D. et al. (2018)	Yes	Yes	Yes	Yes	Yes	Can't tell	Yes	Can't tell	Yes	Moderate
Beatty AL. et al. (2018)	Yes	Yes	Yes	Yes	Yes	Can't tell	Yes	Yes	Yes	Strong
Smith R. et al. (2015)	Yes	Yes	Yes	Yes	Yes	Can't tell	Yes	Yes	Yes	Strong

1. Critical Appraisal Skills Programme. URL: https://casp-uk.net/ [accessed 2019-02-28]
