# Supplementary material for: Barriers to and Facilitators of Technology in Cardiac Rehabilitation and Self-Management: Systematic Qualitative Grounded Theory Review
Source: J Med Internet Res. 2020 Nov 11;22(11):e18025. doi: 10.2196/18025 (PMC7688378; doi:10.2196/18025)
Supplement: Multimedia Appendix 4 [file jmir_v22i11e18025_app4.pdf]

Overview of the studies included in the final review.

Name	Participants	System	Research methodology	Findings
Dithmer et al [1]	Patients in a rehabilitation program diagnosed with heart failure, myocardial infarction, or angina pectoris.	"The heart game" is an app prototype developed for Android running tablets. It presents heart patients with a game-like challenge every day and is designed to be played by a two-person team. It was designed to be used soon after discharge from the hospital and when the patient begins the rehabilitation process.	The prototype has been developed through a user-driven process, and a triangulation of data collection techniques was applied. Evaluation was based on log files of app usage followed by qualitative interviews with 10 patients and 6 teammates.	Inclusion of a close relative or spouse in the game motivated the patients to perform rehabilitation activities. Gamification design principles engaged the users.
Yehle et al [2]	Patients with CHD[a] and their informal caregivers from cardiopulmonary rehabilitation	Two food decision support systems, web based: Food for the Heart and mobile based: Mobile Magic Lens, were	Three focus group sessions with 20 patients with CHD and 7 informal caregivers. During the focus group	Five themes emerged: decreasing carbohydrate intake and portion size are common challenges, clinician and social

	clinic in Indiana, serving predominantly rural-based population.	developed to aid in daily dietary choices.	sessions, participants were asked about their favorite foods, dietary changes made since CHD diagnosis, challenges in making dietary changes, and ways of overcoming these challenges. Content analysis of qualitative data was performed to find themes through a grounded theory approach.	support makes dietary adherence easier, the systems could make meal planning and adherence less complicated, the systems helped save time and assist healthy choices, and additional features would be required to make tools more comprehensive.
Villalba et al [3]	Participants included those who were diagnosed with heart failure or those who had a cardiovascular accident, business managers, and cardiologists.	CUORE is divided into 3 main areas: the user interaction system running on Microsoft's .NET framework, professional interaction through a web-based portal, and a common platform. The sensors and electronics to	A total of 26 people including 10 cardiac patients, 10 cardiologists, and 6 business people were interviewed. The validation comprised 2 phases: first, the system was validated with patients; then, the system was validated with health professionals.	Some patients considered it would be a problem for them to integrate the system into their daily lives. Some felt that the app constantly reminded of their sickness. Most patients stated that being remotely monitored increased their feeling of

		monitor patients in their daily routines include a blood pressure cuff, a weight scale, an electrocardiogram /heart rate monitor, and an oxygen saturation monitor.		security and comfort. Education on symptoms and medication was highly valued. Preference is given to continue using the devices they already own.
Jarvis-selinger et al [4]	Participants included cardiac patients, physicians, nurses, and health professionals.	The study aimed at understanding how internet-based platforms could be used to support self-management and communication among patients, physicians, and health professionals.	Semistructured interviews of a total of 48 participants over a period of 6 months was conducted. An iterative approach to data analysis was taken, employing a constant comparative method as a way to explore subjective experience.	Most important feature of the technology was considered to be sharing of patient health records. Majority of the health professionals felt that providing patients with accurate educational resources would be the best use of technology for self-management. Patients preferred face-to-face contact with doctors.
Fischer et al [5]	Participants were diagnosed with	Web-based visualizations for	Usability of the app was tested using	The tool promoted knowledge and

	heart failure and attended the Manukau Super Clinic.	educating patients and promoting behavioral change through interactive web graphics to visualize relationships between lifestyle, symptoms, patient parameters, and the disease.	surveys. The effectiveness of the app was evaluated through semistructured interviews with 18 participants.	understanding of illness and associated symptoms, thereby alleviating the distress and influencing self-management and behavior change.
Pfaeffli et al [6]	Participants included patients diagnosed with CVDs[b] and CR[c] nurses.	Mobile SMS and brief video vignettes through participant website.	Intervention was developed with patient input using the following steps: conceptualization, formative research, pretesting, and pilot testing. Interviews with 38 CR patients were conducted after the CR program. Interview analysis used thematic approach.	The mHealth[d] format was considered to be particularly useful for patients who found it difficult to attend center-based CR. Older participants viewed technology as a barrier because of unfamiliarity.
Katalinic et al [7]	Participants included patients of stroke,	Two home telehealth technologies (the	A total of 102 patients were involved in the study.	Apart from technical issues such as poor broadband and

	pediatric palliative, brain injury, and cardiac coaching.	Intel Health Guide and the Apple iPad) were trialed by 4 clinical services. The Intel Health Guide for cardiac coaching services and iPad for pediatric palliative care, the stroke and brain injury rehabilitation services.	Intel health guide and iPad were given to use for 3 months. Satisfaction surveys were used to assess the usability and usefulness of videoconferencing and home telehealth devices and clinical advantages of using technology.	connectivity, Telehealth was found to play a useful role in improving access to services, especially for people who lived in rural areas. Both clinicians and patients readily accepted new technology; however, usability and ease of use are crucial in ensuring acceptance of technology.
Antypas and Wangberg [8]	Participants attending the CR program.	Website based on open source content management framework Drupal. It consisted of profile page; activity calendar; a discussion forum; and general information about cardiac disease, training, and symptoms. Users	Conducted a focus group with 11 participants (3 women and 8 men) of a CR program. Thematic analysis was used to identify and analyze transcribed data.	Seven themes were identified: social, motivation, integration into everyday life, information, planning, monitoring and feedback, and concerns and potential problems.

		receive feedback regarding their level of activity.		
Geurts et al [9]	This study involved patients with cardiac condition, cardiologists, and HCI[e] experts for different stages of data collection.	The Back on Bike system consists of a mobile- and browser-based app that monitors cycling efforts of CR patients along with a dashboard for the medical staff.	Observations and contextual inquiries were held in the rehabilitation center with a physiotherapist. Followed by a co-design workshop with 4 HCI experts and 1 cardiologist in training nine patients participated in a field study, and results for 4 of the 9 patients are described in detail in this paper.	The system reassured patients that they were cycling at the right heart rate zone. The system also encouraged healthy people accompanying the patients to adapt their pace to the patient's safe zone. The app reduced fear as it supported patients to cycle with others. Supportive messages and predefine routes motivated them to complete a tour.
Buys et al [10]	Study patients were recruited from a supervised phase 2 ambulatory CR program, 2 community based	The study aimed at understanding current technology usage of patients with CVD. Survey questions related to following	A technology usage questionnaire was completed by 310 patients. The questions were related to patients' characteristics,	Patients were interested in CR support through mobile or internet. Patients reported interest in virtual-based and game

	phase 3 CR programs, and adult congenital heart disease clinic.	technologies: mobile phone, internet, computer games, heart rate monitor, and physical activity monitor.	current technology usage, and patients' interests and needs from a technology-based virtual CR intervention. Data analysis was conducted using a statistical software.	based CR. Advice on exercise ideas, opportunities, diet, and stress received rating for including in technology. Technology should be designed for all ages including men and women.
Cornet et al [11]	Participants diagnosed with heart failure and their informal caregivers.	Engage is a mHealth system designed for mobile or tablet devices to be used by patients or informal caregivers.	15 participants used Engage for 30 days. Two usability studies, task based and scenario based, generated a set of findings, and design guidelines were proposed by triangulating the complementary results from task-based tests, scenario-based evaluation, and quantitative instruments.	Participants were concerned about adding a device on top of what they already have. Participants desired a simpler interface; there is a need to consider affective design and individual differences, in addition to technical usability and performance requirements.
Banner et al [12]	Participants were patients with	The vCRP[f] was designed to mimic a	78 cardiac participants were	Five themes were identified:

	acute coronary syndrome or following a revascularization.	standard hospital-based CRP. It includes web-based intake forms; scheduled one-on-one chat sessions with the program nurse, case manager, exercise specialist, and dietician; weekly education sessions; and data capture for the exercise stress test and blood test results.	enrolled in the study. Control group received routine care from their primary care provider, and intervention group received an orientation to the vCRP. The program lasted 4 months in duration and a final semistructured interview was undertaken with 22 participants. Evaluation included descriptive analysis of the data and thematic coding.	accessibility, making healthy choices, surveillance, barriers to participation, and perceptions of vCRP. Participants reported increased awareness and motivation to manage their health condition. Poor computer literacy was identified as a barrier.
Baek et al [13]	User research was conducted on cardiac patients and doctors at a tertiary general university hospital located in the Seoul	An mHealth mobile phone app was designed using a mock-up tool. The app provides health information, health questionnaire, self-management, and dairy.	Three types of user research and user experience investigations including surveys and interviews with 35 patients, focus group interviews with doctors, and a	Top 3 items that patients thought important included exercise, dietary control, and weight management. The commonly required features were easy app use, up-to-date

	metropolitan area of South Korea.		usability test were conducted. Evaluation was carried out by analyzing the opinions of doctors using the card sorting method, and interview transcripts were analyzed using the constant comparative method.	information on health, self-assessment, current health status, and communication with doctors.
Salvi et al [14]	Patients who had experienced a cardiac event were selected and analyzed for suitability by physicians involved in the project.	The GEx, General exercise, system is composed of 3 main parts: the Mobile station, for monitoring physical exercise and providing live guidance during exercise sessions; the Patient station, which acts as a collector and gateway of patients' data and is responsible for	A randomized controlled trial was conducted with 118 participants to compare mobile-based rehabilitation, 55 patients versus standard care, and 63 patients. User acceptance and perceived usefulness were measured with a questionnaire inspired by the Technology Acceptance Model.	Educational level about heart-related health improved more in the intervention group than the control. Exercise habits at 6-month follow-up also improved.

		delivering educational content to the user; and the Professional station, a web-based app which is used by doctors to prescribe and tailor each exercise program, visualize patient progress, and be alerted in case of problems.		
Beatty et al [15]	Patients from a cardiology clinic who were eligible for CR were the participants.	A mobile app was designed to be used as a tool for home CR and includes physical activity goal setting, logs for physical activity, and health measures.	A total of 13 participants completed the System Usability Scale, rated likelihood to use the mobile app, questionnaires on mobile app use, and participated in a semistructured interview.	There was a desire for introductory training. Family and peer support were reported to influence mobile technology use. Participants desired ease of use and simplicity.
Smith et al [16]	Cardiac patients, physicians, and accredited social health activists.	Assessing the potential for using mHealth and mobile phone usage.	15 participants were involved in semistructured interviews over a	Challenges of CVD management were stated as poor patient disease knowledge,

			period of 6 weeks. Evaluation involved thematic analysis of the interviews.	usability, and lifestyle. Family support, knowledge support, health work, and physician support are considered motivating.

[a]CHD: coronary heart disease.

[b]CVD: cardiovascular disease.

[c]CR: cardiac rehabilitation.

[d]mHealth: mobile health.

[e]HCI: human-computer interaction.

[f]vCRP: virtual cardiac rehabilitation program.

1. Dithmer M, Rasmussen JO, Grönvall E, Spindler H, Hansen J, Nielsen G, Sørensen SB, Dinesen B. “The Heart Game”: Using Gamification as Part of a Telerehabilitation Program for Heart Patients. Games Health J [Internet] Mary Ann Liebert Inc.; 2016;5(1):27–33. Available from: https://www.scopus.com/inward/record.uri?eid=2-s2.0-84993661525&doi=10.1089%2Fg4h.2015.0001&partnerID=40&md5=3359663ef842f7cd79c79c5fd6589fc6
2. Yehle KS, Chen AMH, Plake KS, Yi JS, Mobley AR. A qualitative analysis of coronary heart disease patient views of dietary adherence and web-based and mobile-based nutrition tools. J Cardiopulm Rehabil Prev [Internet] 2012;32(4):203–209. Available from: https://www.scopus.com/inward/record.uri?eid=2-s2.0-84866041308&doi=10.1097%2FHCR.0b013e31825b4e6a&partnerID=40&md5=48174eb20474f124643783ab58ab0f31
3. Villalba E, Peinado I, Arredondo MT. Self care system to assess cardiovascular diseases at home. Lect Notes Comput Sci [Internet] 2009;5615:248–257. Available from: https://www.scopus.com/inward/record.uri?eid=2-s2.0-70350329235&doi=10.1007%2F978-3-642-02710-9_28&partnerID=40&md5=4dde04ce0ae39566561945bd85ce959e
4. Jarvis-selinger S, Bates J, Araki Y, Lear SA. Internet-Based Support for Cardiovascular Disease Management. 2011;2011. [doi: 10.1155/2011/342582]
5. Fischer S, Wünsche BC, Cameron L, Morunga ER, Parikh U, Jago L, Müller S. Web-based Visualisations Supporting Rehabilitation of Heart Failure Patients by Promoting Behavioural Change. Proc Thirty-Fourth Australas Comput Sci Conf -

Vol 113 [Internet] Darlinghurst, Australia, Australia: Australian Computer Society, Inc.; 2011. p. 53–62. Available from: http://dl.acm.org/citation.cfm?id=2459296.2459303

6. Pfaeffli L, Maddison R, Whittaker R, Stewart R, Kerr A, Jiang Y, Kira G, Carter K, Dalleck L. A mHealth cardiac rehabilitation exercise intervention: findings from content development studies. BMC Cardiovasc Disord [Internet] 2012;12(1):36. PMID:22646848
7. Katalinic O, Young A, Doolan D. Case study: The interact home telehealth project. J Telemed Telecare [Internet] 2013;19(7):418–424. Available from: https://www.scopus.com/inward/record.uri?eid=2-s2.0-84890210940&doi=10.1177%2F1357633X13506513&partnerID=40&md5=28a8e317ec057b3c74f048eb12f98d9f
8. Antypas K, Wangberg SC. Combining users' needs with health behavior models in designing an internet- and mobile-based intervention for physical activity in cardiac rehabilitation. J Med Internet Res [Internet] 2014;16(1). Available from: https://www.scopus.com/inward/record.uri?eid=2-s2.0-84893602316&doi=10.2196%2Fresprot.2725&partnerID=40&md5=6cbff99e0af867fe21b98649785c9b8b
9. Geurts E, Haesen M, Dendale P, Luyten K, Coninx K. Back on Bike: The BoB Mobile Cycling App for Secondary Prevention in Cardiac Patients. Proc 18th Int Conf Human-Computer Interact with Mob Devices Serv [Internet] New York, NY, USA: ACM; 2016. p. 135–146. [doi: 10.1145/2935334.2935377]
10. Buys R, Claes J, Walsh D, Cornelis N, Moran K, Budts W, Woods C, Cornelissen VA. Cardiac patients show high interest in technology enabled cardiovascular rehabilitation. BMC Med Inform Decis Mak [Internet] B.E.R.G. Bica, Department of Internal Medicine, Hospital Universitario Clementino Fraga Filho, Universidade Federal do Rio de Janeiro, Brazil. E-mail: rios.belena@gmail.com: BioMed Central Ltd.; 2016;16(1). Available from: https://www.scopus.com/inward/record.uri?eid=2-s2.0-84978706350&doi=10.1186%2Fs12911-016-0329-9&partnerID=40&md5=32bf18f647518a0bc17b28824e5da23e
11. Cornet VP, Daley CN, Srinivas P, Holden RJ. User-centered evaluations with older adults: Testing the usability of a mobile health system for heart failure self-management. Proc Hum Factors Ergon Soc 2017;2017-Octob:6–10. [doi: 10.1177/1541931213601497]
12. Banner D, Lear S, Kandola D, Singer J, Horvat D, Bates J, Ignaszewski A. The experiences of patients undertaking a "Virtual" cardiac rehabilitation program. Stud Heal Technol Informatics [Internet] IOS Press; 2015;209:9–14. Available from: https://www.scopus.com/inward/record.uri?eid=2-s2.0-84937135399&doi=10.3233%2F978-1-61499-505-0-9&partnerID=40&md5=36c7576f6c1e43451ad5076492c924f4
13. Baek H, Suh JW, Kang SH, Kang S, Lim TH, Hwang H, Yoo S. Enhancing user experience through user study: Design of an mhealth tool for self-management and care engagement of cardiovascular disease patients. J Med Internet Res 2018;20(2):1–12. [doi: 10.2196/cardio.9000]

14. Salvi D, Ottaviano M, Muuraiskangas S, Martínez-Romero A, Vera-Muñoz C, Triantafyllidis A, Cabrera Umpiérrez MF, Arredondo Waldmeyer MT, Skobel E, Knackstedt C, Liedes H, Honka A, Luprano J, Cleland JGF, Stut W, Deighan C. An m-Health system for education and motivation in cardiac rehabilitation: the experience of HeartCycle guided exercise. J Telemed Telecare [Internet] N.E. Aikawa, Faculdade de Medicina, Universidade de Sao Paulo, Brazil: SAGE Publications Ltd; 2018;24(4):303–316. Available from: https://www.scopus.com/inward/record.uri?eid=2-s2.0-85026843486&doi=10.1177%2F1357633X17697501&partnerID=40&md5=90310e58712beda42002bbdc6c7f0f4a
15. Beatty AL, Magnusson SL, Fortney JC, Sayre GG, Whooley MA. VA fitheart, a mobile app for cardiac rehabilitation: Usability study. J Med Internet Res [Internet] Journal of Medical Internet Research; 2018;20(1). Available from: https://www.scopus.com/inward/record.uri?eid=2-s2.0-85041088485&doi=10.2196%2Fhumanfactors.8017&partnerID=40&md5=d89c70d62d7ef46fcb628eb52f3a5b32
16. Smith R, Menon J, Rajeev JG, Feinberg L, Kumar RK, Banerjee A. Potential for the use of mHealth in the management of cardiovascular disease in Kerala: a qualitative study. BMJ Open [Internet] 2015;5(11):e009367. Available from: https://www.scopus.com/inward/record.uri?eid=2-s2.0-85019494444&doi=10.1136%2Fbmjopen-2015-009367&partnerID=40&md5=db4e68f5f16c98c4723efda89154321c
